# Expectations predict early pregnancy-related symptom burden

**DOI:** 10.1038/s41598-026-57961-w

**Published:** 2026-06-16

**Authors:** Meike Shedden-Mora, Verena Schymanski, Renja Albers, Belana Beth, Susan Garthus-Niegel, Alia J. Crum, Lisa Autzen

**Affiliations:** 1 Institute for Clinical Psychology and Psychotherapy, Department of Psychology, Medical School Hamburg, Hamburg, Germany; 2 Department of Psychosomatic Medicine and Psychotherapy, University Medical Center Hamburg-Eppendorf, Hamburg, Germany; 3 Institute for Systems Medicine (ISM), Faculty of Human Medicine, Medical School Hamburg, Hamburg, Germany; 4 Institute and Policlinic of Occupational and Social Medicine, Faculty of Medicine, Technische Universität Dresden, Dresden, Germany; 5 Department of Childhood and Families, Norwegian Institute of Public Health, Oslo, Norway; 6 Department of Psychology, Stanford University, Stanford, USA

**Keywords:** Early pregnancy, Symptom expectations, Pregnancy-related symptom burden, Symptom perception, Diseases, Health care, Medical research, Psychology, Psychology, Risk factors, Signs and symptoms

## Abstract

Early pregnancy is commonly accompanied by a wide range of symptoms, yet psychological determinants of pregnancy-related symptom burden remain poorly understood. Evidence from other medical contexts suggests that expectations may contribute to symptom burden. This prospective study examined whether first-trimester symptom expectations predict pregnancy-related symptom burden and disability at the beginning of the second trimester. In the PrExpect study, pregnant women up to the 10th gestational week completed online questionnaires assessing pregnancy-related symptoms, symptom expectations, psychological variables, and pregnancy characteristics, with follow-up at the beginning of the second trimester. Hierarchical regression models identified predictors of symptom burden and disability. Bidirectional temporal associations between symptoms and expectations were examined using a cross-lagged panel model. Exploratory analyses assessed pregnancy-related mindsets. A total of 347 women provided baseline data, and 227 completed follow-ups. Symptom burden was high and increased across the first trimester. Stronger baseline symptom expectations consistently predicted greater symptom burden and disability at follow-up. Cross-lagged analyses indicated bidirectional associations between expectations and symptoms over time. Several negative pregnancy-related mindsets were associated with higher symptom burden. These findings identify symptom expectations as a psychological correlate of early pregnancy-related symptom burden and disability, suggesting that symptom expectations may represent a promising target for future intervention studies to support well-being in early pregnancy.

**Supplementary Information:**

The online version contains supplementary material available at 10.1038/s41598-026-57961-w.

## Introduction

For many women, early pregnancy is an intense and closely monitored period, characterized by heightened attention to bodily sensations, anticipation of milestones, and adjustment to a new life context.^1^ Physical symptoms are the norm rather than the exception, with nausea, vomiting, fatigue, and back or pelvic pain among the most commonly reported.^2^ In fact, 88% report at least two symptoms, while only 2% report having no symptoms at all.^3^ Pregnancy-related symptoms are not only associated with high healthcare costs and costs due to loss of workforce,^4,5^ they reduce well-being and mental and physical quality of life.^5,6^ The intensity of nausea and vomiting is associated with increased postpartum depression and anxiety levels.^7,8^ Pregnancy-related symptoms can constitute a substantial stressor. Psychological distress, in turn, is consistently associated with a higher risk of adverse maternal, perinatal, and child outcomes, including increased risk of preterm birth, greater use of epidural analgesia during childbirth, postpartum affective symptoms, low birth weight, and even altered fetal brain development, including changes in brain biochemistry, hippocampal growth, and cortical folding. ^9–11^ Notably, symptoms beyond nausea and vomiting, such as fatigue, sleep problems, or pain, receive surprisingly little scientific attention.

Regarding the aetiology of symptoms, hormonal processes – particularly human chorionic gonadotropin (hCG) – are implicated in nausea and vomiting.^12^ While nausea and vomiting have been associated with more favourable pregnancy outcomes,^13^ overly high hCG concentrations and extreme nausea and vomiting, known as hyperemesis gravidarum, have been associated with fetal growth delay and risk of preeclampsia.^14^ Importantly, hCG levels do not sufficiently account for the marked interindividual variability in symptom burden.^12^.

From a biopsychosocial perspective, physical symptoms are not mere reflections of pathophysiology.^15,16^ Current aetiological models conceptualize physical symptoms emerging from dynamic interactions between biological, psychological, and social factors.^15^ In the vulnerability-stress model for persistent physical symptoms, known predisposing factors include sociodemographic factors such as female sex, and low socioeconomic status, biomedical factors such as chronic medical conditions and certain (epi-)genetic profiles, and psychosocial factors such as early adverse experiences, and negative affectivity. Symptom burden is maintained by biomedical processes such as chronic inflammation and psychological processes including negative expectations, anxiety, heightened attention to bodily sensations, and a self-concept of bodily weakness.^15,17^.

Adopting this perspective implies that pregnancy-related symptoms are not merely a result of hormonal levels. Early pregnancy symptoms might be shaped by similar psychosocial factors. Importantly, many of these factors are modifiable and could be addressed through psychosocial interventions.^15^ Few studies have investigated psychosocial predictors of pregnancy-related symptom burden. In a cross-sectional analysis of the HAPPY study,^18^ high hCG levels, current and past depressive symptoms, younger age, and multiparity were associated with higher levels of nausea and vomiting at 12 to 14 weeks of gestation.^12^ Another cross-sectional study of 570 women in their 11th to 14th gestational week found younger age and higher household income to be associated with nausea and vomiting, but no further psychological factors were investigated.^19^ In a small prospective study of 59 women, anxiety and insomnia at 7 to 9 weeks of gestation predicted nausea and vomiting at 11 to 13 weeks.^20^ Goodwin argues that psychological factors, alongside physiological triggers (i.e., hCG levels), can amplify or sustain nausea and vomiting through learned or mood-related pathways.^21^ However, prospective research examining psychological predictors of pregnancy-related symptom burden remains scarce and has largely been limited to relatively small samples and specific symptoms such as nausea and vomiting. ^20^

Expectations have been identified as strong predictors of symptom burden and treatment outcomes across many physical and mental health conditions, including chronic pain and other persistent somatic symptoms, depression, and surgical procedures.^22–24^ In pregnancy, women’s expectations may contribute to differences in pregnancy-related symptom burden. Although little research has investigated the role of symptom-related expectations in early pregnancy, broad evidence has shown that women’s expectations predict the quality of their birth experience.^25^ Women with positive expectations rate their births as more satisfying and as less painful,^26^ whereas women with higher expectations of labour pain and more fear regarding delivery are more likely to report negative birth experiences and to request a caesarean Sects.^27,28^.

Beyond specific symptom expectations, broader symptom and body mindsets - core beliefs about the meaning of symptoms and the body^29^- might influence symptom experiences in early pregnancy. People with more adaptive body and symptom mindsets, such as viewing the body as capable or side effects as positive signals, tend to report more favourable health outcomes.^29–31^ In pregnancy, mindsets favouring a more natural versus medically assisted birth are associated with better birth experiences and well-being.^32,33^ An extensive longitudinal study showed that a prenatal natural mindset was linked to a likelihood of a low-intervention birth and a more positive birth experience, predicting higher early postpartum well-being, lower postpartum depression and post-traumatic stress symptoms at 8 weeks, as well as better mother-infant bonding at 6 months.^34^ However, pregnancy-related symptom mindsets have not yet been studied.

The current PrExpect study examined whether women’s expectations predict the occurrence of early pregnancy-related symptom burden. We hypothesized that symptom expectations in the first trimester would predict pregnancy-related symptom burden and symptom-related disability at the beginning of the second trimester over and above existing baseline symptoms. Moreover, the role of depressive symptoms, anxiety, interoceptive sensitivity, symptom-proneness, self-efficacy, optimism, and previous experiences as predictors for pregnancy-related symptom burden and disability in the first trimester was investigated. We additionally explored the association of pregnancy-related symptom mindsets with symptom burden.

## Methods

### Participants

Pregnant women up to the 10th gestational week were invited to participate. Pregnancy was confirmed by self-testing for hCG concentration in urine, blood test, or gynaecological ultrasound. Women were recruited via gynaecological practices, fertility centres, via social media (Instagram, Facebook, influencers on pregnancy-related topics), and via German online forums about parenthood (e.g., Babyclub). Participants could participate in a draw to win a 20€ voucher for a German drugstore company.

A pragmatic a priori sample size target of *N* = 200 women with complete data at both assessment points was defined based on feasibility considerations and to ensure a sufficiently representative sample and adequate stability of the planned multivariate predictive models. Anticipating an attrition rate of approximately 33% due to pregnancy loss and non-response, we aimed to recruit an initial sample of *N* = 300 women.

### Study design and recruitment

The PrExpect study was a prospective observational study with two assessment points. The study received ethical approval from the ethics committee of the Medical School Hamburg, Germany, on 02/22/2023 (Approval No. MSH-2023/232). All methods were performed in accordance with relevant guidelines and regulations and in accordance with the Declaration of Helsinki for research involving human participants. Reporting followed the STROBE guidelines.^35^ It was pre-registered at the Open Science Framework (https://osf.io/52y4u).

Pregnant women up to the 10th week of pregnancy were invited to complete an online survey using Unipark (Questback GmbH). The second assessment took place at the start of the second trimester, between the 13th and 14th week, at latest in the 20th week, with a minimum interval of three weeks between assessments. Women who did not respond to the first invitation for the second assessment were reinvited twice after one and two weeks. After receiving written study information, all participants provided informed consent prior to study participation. Following, they completed the online questionnaires.

### Assessment

#### Primary and secondary outcomes

The primary outcome was pregnancy-related symptom burden at the start of the second trimester, defined as subjectively perceived symptom burden due to the pregnancy. ^2,17^ It was assessed using 28 common symptoms from the Pregnancy Symptom Inventory (PSI).^2^ We added hypersalivation as a symptom occurring particularly in early pregnancy.^36^ In the absence of a validated German version, all 29 items were translated. Each symptom was rated on a 3-point scale from ‘no burden’ to ‘high burden’. A sum score was calculated with higher values indicating higher symptom burden.

As a secondary outcome, symptom-related disability was assessed with the Pain Disability Index (PDI) in the validated version for general symptoms.^37^ The PDI measures disability in 7 areas of life on a 10-point scale from ‘no disability’ to ‘complete disability’. A sum score was calculated with higher values indicating higher disability.

#### Predictors

Predictors assessed in the first trimester included sociodemographic and pregnancy-related variables, psychological factors, symptom-related factors, and expectations, as detailed below.

As *sociodemographic variables*, age, marital status, partnership, education (in years), employment status, and current sick leave were assessed. *Pregnancy-related* variables included the type of pregnancy detection (self-test, blood test, ultrasound), assumed week of pregnancy, due date, previous pregnancies and births, previous miscarriages, pregnancy after fertility treatment, whether the pregnancy was planned, and medication with potential influence on the pregnancy. The overall experience with previous pregnancies was assessed with one item on a 5-point scale from ‘very negative’ to ‘very positive’. Unfortunately, due to a technical survey error, data on the number of previous pregnancies and the experience were partly invalid and could therefore not be included in the main analyses.

Further, the attitude towards the pregnancy was assessed with one item on a 6-point scale from ‘negative’ to ‘positive’. Women were asked how well informed they felt on a 6-point scale from ‘not at all’ to ‘very well’. Finally, women were asked whether they had already disclosed their pregnancy towards others (co-parent, family, friends, work environment, further social network).

As psychological factors, *depressive symptoms* during the past 2 weeks were assessed using the Patient Health Questionnaire (PHQ-9)^38^ with 9 items on a 4-point scale ranging from ‘not at all’ to ‘nearly every day’. *Pregnancy-related anxiety* was measured with two items from the Pregnancy Anxiety Questionnaire - revised (PRAQ-R2)^39^ assessing worries about birth on a 5-point scale from ‘absolutely not relevant’ to ‘very relevant’.

*Self-efficacy*,* optimism*,* and pessimism* were measured with the Self-Efficacy, Optimism and Pessimism Questionnaire (SWOP-K9)^40^ using 5 items for self-efficacy and 2 items each for optimism and pessimism on a 4-point scale ranging from ‘not true’ to ‘true’.

As symptom-related factors, *baseline symptoms* were assessed with the adapted PSI,^2^ as described above. The extent of *proneness to four common pregnancy symptoms* (nausea, vomiting, dizziness, fatigue) was assessed on a 4-point scale ranging from ‘not at all’ to ‘very much’.

*Sensitivity and attention to interoceptive signals* was measured with the Interoceptive Sensitivity and Attention Questionnaire (ISAQ).^41^ Eight items from the two subscales “attention to unpleasant physical sensations” and “difficulty disengaging from unpleasant physical sensations” were rated on a 5-point scale from ‘strongly disagree’ to ‘strongly agree’.

*Expectations* regarding pregnancy-related symptom burden were assessed with an adapted version of the same PSI used to assess pregnancy-related symptom burden, specifically developed in the context of the PrExpect study (PSI-expect).^2^ Women were asked how much symptom burden they expected regarding the 29 symptoms in the next four weeks on the 3-point scale.

Two pregnancy-specific *mindset* items were developed after pre-registration, informed by prior work on body and symptom mindsets.^29–31^ A symptom mindset - seeing pregnancy symptoms as reassuring signs - was assessed with the item “Pregnancy symptoms are a positive sign that everything is OK with the pregnancy.”, rated on a 6-point scale from ‘strongly disagree’ to ‘strongly agree.’ A body mindset, reflecting the perception of the body as capable, was measured with the item “I feel that my body is not strong enough to cope with the pregnancy.”, rated on the same scale and reverse-coded. Given their exploratory nature, items were analysed separately.

### Statistical analyses

For all analyses with two-sided testing, a probability of error of α = 0.05 is assumed. All model assumptions are checked prior to the analyses. Analyses were conducted using R Studio 2025.05.1.

The online survey used a forced-response format; therefore, no item-level missing data occurred among completed assessments. Plausibility checks were conducted prior to analyses, and no participants needed to be excluded due to implausible response patterns.

To analyse predictors of pregnancy-related symptoms at the start of the second trimester, a hierarchical multiple linear regression model was calculated. The primary outcome was pregnancy-related symptom burden (PSI); the secondary outcome was symptom-related disability (PDI). Assumptions of linear regression, including linearity, normality and homoscedasticity of residuals, multicollinearity, and influential outliers, were checked and considered acceptable. Residuals were approximately normally distributed and plots indicated no major deviations from homoscedasticity or linearity. No problematic multicollinearity was observed (all Variance Inflation Factors < 2.0). No influential outliers were identified (Cook’s distances < 0.08).

Predictors were entered hierarchically based on theoretical considerations and prior research. First, pregnancy-related factors were entered, followed by psychological factors and symptom-related factors, including baseline symptom burden. Finally, symptom expectations were added to examine their incremental predictive value beyond established correlates of pregnancy-related symptom burden. Variables showing significant bivariate associations with the outcomes were included in four steps: (1) pregnancy-related factors, (2) psychological factors, (3) symptom-related factors, and (4) symptom expectations.

The two mindsets were analysed separately and correlated with pregnancy-related symptoms at the start of the second trimester.

To examine the temporal association between symptom expectations and pregnancy-related symptoms, we estimated a two-wave cross-lagged panel model (CLPM)^42^ in an additional analysis that was not preregistered. The weighted least squares mean and variance adjusted (WLSMV) estimator was used as a robust estimator given slight deviations from normality in the study variables.^43^.

The model included autoregressive (T0→T1) paths for each construct, the two cross-lagged paths between constructs, within-wave covariances at T0 and T1, and residual variances. Equality of the two cross-lagged paths was evaluated with a Wald test. Standardized coefficients (std.all) are reported.

## Results

### Sample characteristics

*N* = 347 pregnant women completed the first assessment up to the 10th gestational week. Of 253 women who accessed the link for the second assessment, 25 reported pregnancy loss; no further information was available for women who did not access the follow-up link. One participant who completed only the gestational week item and provided no further data was excluded. *N* = 227 (62.2%) completed the second assessment at the start of the second trimester and constituted the sample for all analyses. Women who completed the second assessment did not differ from those who only completed the first assessment regarding age, education, history of miscarriage, pregnancy attitude, baseline pregnancy-related symptom burden, symptom expectations, depressive symptoms, or pregnancy-related anxiety (all *p* > .05). However, women completing the second assessment were further advanced in their pregnancy at baseline (gestational week: *M* = 7.66, *SD* = 1.78 vs. *M* = 7.10, *SD* = 1.63), *t* = -2.95, *p* = .003, and more likely to have undergone fertility treatment (20.1% vs. 11.7%), χ² = 4.06, *p* = .044.

Table 1 summarizes demographics and pregnancy characteristics. Participants were on average 31 years old and were in their 4th to 10th week of pregnancy.

**Table 1:** Sample demographics.

Variable	Sample *n* (%)/m(SD)
Pregnancy week at baseline, m (SD), range	7.66 (1.78); 4–10
Week 4	8 (3.5%)
Week 5	22 (9.7%)
Week 6	40 (17.6%)
Week 7	32 (14.1%)
Week 8	36 (15.9%)
Week 9	45 (19.8%)
Week 10	44 (19.4%)
Pregnancy week at second assessment, m (SD), range	13.98 (1.26); 13–20
Week 13	98 (43.2%)
Week 14	77 (33.9%)
Week 15	32 (14.1%)
Week 16	11 (4.8%)
Week 17	4 (1.8%)
Week 18	1 (0.4%)
Week 19	1 (0.4%)
Week 20	3 (1.3%)
Age, m (SD), range	30.81 (4.42); 19–41
Gender, female	227 (100%)
Nationality	
German	219 (96.5%)
Other	8 (3.5%)
Marital status	
Single	86 (37.9%)
Married	136 (59.9%)
Separated	1 (0.4%)
Other	4 (1.8%)
In a relationship	219 (96.5%)
Education ≥ 12 years	194 (85.5%)
Employment status	
Employed	199 (87.7%)
Unemployed	28 (12.3%)
Current sick leave	
No	188 (82.8%)
Yes, up to 2 weeks	17 (7.5%)
Yes, 2–4 weeks	17 (7.5%)
Yes, more than 4 weeks	5 (2.2%)
Planned pregnancy	200 (88.1%)
Fertility treatment	46 (20.3%)
Previous pregnancies (*n* = 180)*	
None	122 (67.8%)
1	29 (16.1%)
2	21 (11.7%)
3	4 (2.2%)
4	2 (1.1%)
5	2 (1.1%)
Quality of previous pregnancy experience (*n* = 58)*	
1 (very negative)	8 (13.8%)
2	9 (15.5%)
3	18 (31.0%)
4	15 (25.9%)
5 (very positive)	8 (13.8%)
History of miscarriage(s)	53 (23.3%)
Medication intake	114 (50.2%)
Medication intake that can affect pregnancy	42 (18.5%)
Pregnancy disclosure	
To co-parent	202 (88.2%)
To family members	134 (58.5%)
To close friends	140 (61.1%)
To employer	80 (34.9%)
To broader social network	16 (7.0%)
Attitude towards pregnancy	
1 (negative)	0 (0%)
2	0 (0%)
3	12 (5.3%)
4	31 (13.7%)
5	59 (26.0%)
6 (positive)	125 (55.1%)
Feeling well informed about the pregnancy	
0 (not at all)	2 (0.9%)
1	5 (2.2%)
2	24 (10.6%)
3	76 (33.5%)
4	65 (28.6%)
5 (very well)	55 (24.2%)

*n* = 227. *Data only available for *n* = 180 women due to a technical survey error.

Most lived with a partner (96.5%) and were married (59.8%). Most held at least 12 years of education (84.7%), were employed (87.8%), and were not on sick leave (82.2%). Fertility treatment had been used by 20.1%; 23.1% reported a previous miscarriage. Half were taking medication (49.3%), of which 18.8% had potential pregnancy impact. As mentioned in the methods section, due to a technical survey error, data on the number of previous pregnancies and the experience were only available for *n* = 180 women. Therefore, the variables could not be included in the prediction analyses.

For all scales, descriptive statistics and internal consistencies (Cronbach’s α) are reported in the supplement (Suppl. Table S1). Internal consistencies were acceptable to good (Cronbach’s α = 0.65–0.88), except for the two ISAQ subscales, which showed lower internal consistencies (attention: α = 0.60; difficulty disengaging: α = 0.49).

### Pregnancy-related symptoms

The prevalences of single symptoms and overall pregnancy-related symptom burden are shown in Table 2. In the first trimester, only one woman (0.4%) reported no symptoms, and 12 women (5.2%) reported less than 5 symptoms. At the start of the second trimester, all women reported symptoms, with only 5 women (2.2%) reporting less than 5 symptoms. On average, women reported 11.6 symptoms at the first and 13.9 symptoms at the second assessment, reflecting a significant increase in symptom burden (*p* < .001). The five most common symptoms at the first and second assessment were tiredness and fatigue (93.8% and 97.4%), painful breasts (77.1% and 68.3%), the frequent urge to urinate (68.3% and 78.4%), nausea (67.4% and 79.7%), and poor sleep (63.9% and 78.4%). The prevalence of 17 of 29 single symptoms increased over time, while only the prevalence of painful breasts significantly decreased over time.

**Table 2:** Prevalence of pregnancy-related complaints in the first and second trimester assessment.

Symptom	First trimester, *n* (%)	Second trimester, *n* (%)	Comparison of prevalences
Tiredness or fatigue	213 (93.8%)	221 (97.4%)	χ² = 2.72, *p* = .099
Breast pain	175 (77.1%)	155 (68.3%)	χ² = 6.22, *p* = .013
Urinary frequency	155 (68.3%)	178 (78.4%)	χ² = 6.82, *p* = .009
Nausea	153 (67.4%)	181 (79.7%)	χ² = 14.58, *p* < .001
Poor sleep	145 (63.9%)	178 (78.4%)	χ² = 16.79, *p* < .001
Shortness of breath	131 (57.7%)	149 (65.6%)	χ² = 4.52, *p* = .034
Taste/smell changes	127 (55.9%)	127 (55.9%)	χ² = 0, *p* = 1.000
Forgetfulness	112 (49.3%)	130 (57.3%)	χ² = 4.38, *p* = .036
Changes in nipples	110 (48.5%)	97 (42.7%)	χ² = 1.58, *p* = .208
Vivid dreams	105 (46.3%)	112 (49.3%)	χ² = 0.49, *p* = .483
Changes in libido	102 (44.9%)	135 (59.5%)	χ² = 10.78, *p* = .001
Dizziness	102 (44.9%)	110 (48.5%)	χ² = 0.68, *p* = .409
Food cravings	94 (41.4%)	124 (54.6%)	χ² = 10.01, *p* = .002
Constipation	91 (40.1%)	128 (56.4%)	χ² = 13.94, *p* < .001
Headache	89 (39.2%)	135 (59.5%)	χ² = 28.12, *p* < .001
Anxiety	88 (38.8%)	88 (38.8%)	χ² = 0, *p* = 1.000
Back pain	87 (38.3%)	118 (52%)	χ² = 11.69, *p* < .001
Dry mouth	80 (35.2%)	81 (35.7%)	χ² = 0, *p* = 1.000
Altered body image	71 (31.3%)	101 (44.5%)	χ² = 13.14, *p* < .001
Sore nipples	64 (28.2%)	67 (29.5%)	χ² = 0.08, *p* = .779
Reflux	59 (26%)	86 (37.9%)	χ² = 12.29, *p* < .001
Hip or pelvic pain	56 (24.7%)	72 (31.7%)	χ² = 3.12, *p* = .077
Vomiting	46 (20.3%)	87 (38.3%)	χ² = 28.07, *p* < .001
Feeling depressed	37 (16.3%)	69 (30.4%)	χ² = 16.02, *p* < .001
Sciatica/pain down the back of your legs	35 (15.4%)	60 (26.4%)	χ² = 10.47, *p* = .001
Restless legs	30 (13.2%)	58 (25.6%)	χ² = 18.23, *p* < .001
Hypersalivation	28 (12.3%)	32 (14.1%)	χ² = 0.24, *p* = .626
Itchy skin	26 (11.5%)	58 (25.6%)	χ² = 19.22, *p* < .001
Leg cramps	20 (8.8%)	22 (9.7%)	χ² = 0.03, *p* = .864
Pregnancy-related symptom burden (PSI), sum (SD)	11.59 (4.56)	13.92 (5.22)	T = -7.85, *p* < .001

*n* = 227. Symptoms are sorted according to prevalence at the first assessment. Comparisons are based on McNemar tests for paired proportions.

### Predictors of pregnancy-related symptoms (primary outcome)

Prior to regression analysis, bivariate correlations between first trimester predictors and pregnancy-related symptom burden at the start of the second trimester were calculated (Suppl. Table S1). Higher pregnancy-related symptom burden was related to stronger symptom expectations (*r* = .67, *p* < .001), more baseline pregnancy-related symptoms (*r* = .60, *p* < .001), more depressive symptoms (*r* = .49, *p* < .001), greater pregnancy-related anxiety (*r* = .28, *p* < .001), greater proneness to symptoms (*r* = .16, *p* = .019), lower positive attitude toward pregnancy (*r* = − .18, *p* = .005), lower self-efficacy (*r* = − .25, *p* < .001), and feeling less informed about the pregnancy (*r* = − .26, *p* < .001).

The hierarchical multiple linear regression model explained 54% of the variance in pregnancy-related symptoms (Table 3). More baseline pregnancy-related symptoms (β = 0.34, [0.22, 0.46], *p* < .001) and stronger symptom expectations (β = 0.45, 95% CI [0.34, 0.56], *p* < .001) uniquely predicted higher pregnancy-related symptom burden at the start of the second trimester; all other variables were not significant after adjustment.

**Table 3:** Predictors of pregnancy-related symptom burden and disability at the beginning of the second trimester (hierarchical multiple linear regression model).

Outcomes	Pregnancy-related symptom burden (PSI)				Symptom-related disability (PDI)			
	β (stand.)	95% CI	t	*p*	β (stand.)	95% CI	t	*p*
Step 1: Pregnancy-related factors	(R^2^_adj_ = 0.07, *p* < .001)				(R^2^_adj_ = 0.03, *p* = .003)			
Positive attitude towards pregnancy	− 0.09	[− 0.19, 0.01]	− 1.76	0.080	− 0.14	[− 0.26, − 0.01]	− 2.14	**0.033**
Feeling well informed about pregnancy	− 0.05	[− 0.15, 0.05]	− 1.02	0.309	0.01	[− 0.12, 0.14]	0.21	0.835
Step 2: Psychological factors	(R^2^_adj_ = 0.28, ΔR² = 0.22, *p* < .001)				(R^2^_adj_ = 0.18, ΔR² = 0.15, *p* < .001)			
Depressive symptoms (PHQ-9)	− 0.01	[− 0.13, 0.12]	− 0.10	0.921	0.19	[0.03, 0.35]	2.34	**0.020**
Pregnancy-related anxiety (PRAQ)	0.07	[− 0.03, 0.17]	1.47	0.143	0.02	[− 0.11, 0.15]	0.32	0.747
Self-efficacy (SWOP-K9)	− 0.03	[− 0.14, 0.08]	− 0.58	0.563	0.07	[− 0.07, 0.22]	1.02	0.308
Optimism (SWOP-K9)	–	–	–	–	− 0.08	[− 0.22, 0.06]	− 1.17	0.242
Pessimism (SWOP-K9)	− 0.00	[− 0.10, 0.10]	− 0.01	0.989	− 0.01	[− 0.14, 0.13]	− 0.08	0.934
Step 3: Symptom-related factors	(R^2^_adj_ = 0.41, ΔR² = 0.14; *p* < .001)				(R^2^_adj_ = 0.20, ΔR² = 0.02, *p* = .024)			
Proneness to symptoms	− 0.00	[− 0.10, 0.10]	− 0.01	0.989	–	–	–	–
Pregnancy-related symptoms (PSI)	0.34	[0.22, 0.46]	5.65	**< 0.001**	0.09	[− 0.07, 0.24]	1.09	0.277
Step 4: Expectations	(R^2^_adj_ = 0.54, ΔR² = 0.13; *p* < .001)				(R^2^_adj_ = 0.25, ΔR² = 0.06, *p* < .001)			
Symptom expectations (PSI-expect)	0.45	[0.34, 0.56]	7.97	**< 0.001**	0.30	[0.16, 0.45]	4.20	**< 0.001**

*n* = 227. Regression coefficients from the final regression model are reported.

### Predictors of symptom-related disability (secondary outcome)

Analogous bivariate correlations and subsequent regression models were calculated for symptom-related disability (Suppl. Table S1). Higher symptom-related disability was related to stronger symptom expectations (*r* = .45, *p* < .001), more depressive symptoms (*r* = .43, *p* < .001), more baseline symptoms (*r* = .36, *p* < .001), greater pregnancy-related anxiety (*r* = .16, *p* = .016), lower positive attitude (*r* = − .21, *p* = .002), feeling less informed (*r* = − .16, *p* = .017), lower optimism (*r* = − .17, *p* = .012), and lower self-efficacy (*r* = − .14, *p* = .033). In the final multivariable regression model, explaining 26% of the variance, a less positive pregnancy attitude (β = −0.14, [− 0.26, − 0.01], *p* = .033), more depressive symptoms (β = 0.19, [0.03, 0.35], *p* = .020), and stronger symptom expectations (β = 0.30, [0.16, 0.45], *p* < .001) predicted greater disability (Table 3).

Additional analyses including gestational week at baseline yielded highly similar results. Pregnancy week was not significantly associated with second-trimester symptom burden or disability and did not meaningfully alter the predictive effects of symptom expectations.

### Temporal relation between expectations and pregnancy-related symptoms

**Fig. 1:**
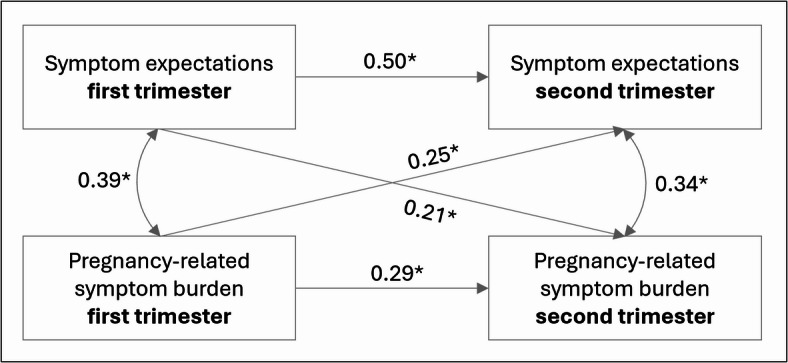
Cross-lagged panel model of symptom expectations and pregnancy-related symptoms from the first to the second trimester. Paths are standardized coefficients (std.all). Solid arrows depict autoregressive (stability) paths; diagonal arrows depict cross-lagged paths. Curved double-headed arrows represent within-wave covariances. *= significant *p*<.05.

The CLPM showed bidirectional cross-lagged effects, with higher symptom expectations in the first trimester predicting more pregnancy-related symptoms at the second-trimester assessment (β = 0.21, SE = 0.06, z = 3.51, *p* < .001), and vice versa (β = 0.25, SE = 0.05, z = 5.26, *p* < .001), each controlling for the corresponding baseline level (Fig. 1). Stability was moderate for symptoms (β = 0.29, SE = 0.07, z = 4.31, *p* < .001) and strong for expectations (β = 0.50, SE = 0.04, z = 12.92, *p* < .001). The model explained 17% of the variance in symptoms and 40% in expectations at the second-trimester assessment. A Wald test indicated that the cross-lagged paths did not differ in magnitude (χ²(1) = 0.99, *p* = .32). The specified model was just-identified (df = 0), resulting in perfect fit indices by definition.

### Mindsets

At the first trimester assessment, women who saw their symptoms as reassuring signs had higher symptom expectations (*r* = .18, *p* = .006), while women who saw their body as capable had lower symptom expectations (*r* = -.26, *p* < .001). The mindset of seeing symptoms as reassuring signs at the first assessment was linked to higher pregnancy-related symptom burden at the beginning of the second trimester (*r* = .20; *p* = .002), but not to disability (*r* = .11, *p* = .092). Seeing one’s body as capable during the first trimester was correlated with lower symptom burden (*r* = -.21, *p* = .001) and disability (*r* = -.20, *p* = .003) at the beginning of the second trimester (see Suppl. Table S2 for a full correlation table).

## Discussion

The PrExpect study examined whether symptom expectations and psychological factors in early pregnancy predict pregnancy-related symptom burden and disability at the beginning of the second trimester. As hypothesized, stronger symptom expectations in the first trimester robustly predicted higher symptom burden beyond baseline symptoms. Other correlated psychological factors, i.e., depressive symptoms, pregnancy-related anxiety, lower self-efficacy, higher pessimism, proneness to symptoms, attitude towards the pregnancy, and feeling informed, did not remain significant after adjustment.

For the secondary outcome, symptom-related disability was predicted by negative pregnancy attitude, higher depressive symptoms, and stronger symptom-related expectations. Expectations consistently emerged as the strongest and most reliable psychological predictor across both outcomes.

Overall, pregnancy-related symptom burden was strikingly high. Women reported an average of 11.6 symptoms in the first trimester, and 13.9 symptoms at the beginning of the second trimester, with fatigue, nausea, poor sleep, urinary frequency, and painful breasts being the most common. Symptoms were already prevalent in very early pregnancy and were unrelated to gestational week, underscoring that symptom burden begins early and extends beyond the most commonly studied symptoms, i.e., nausea and vomiting.

Our findings are consistent with earlier work identifying depressive symptoms^12^ and anxiety^20^ as correlates of pregnancy-related symptoms, although these associations did not persist in multivariable models. Our study did not confirm that younger women report more symptoms.^12,19^.

Notably, this is the first prospective study showing that symptom expectations predict pregnancy-related symptom burden and disability beyond existing symptoms. Although the PSI-expect was adapted from the PSI and therefore assessed expectations regarding the same symptom domains later evaluated as outcomes, expectations prospectively predicted later symptom burden above and beyond baseline symptoms, suggesting that the PSI-expect captured more than concurrent symptom experience alone. This aligns with extensive placebo and nocebo research showing that expectations influence symptom perception and health outcomes.^24,44,45^ The observed bidirectional associations are consistent with frameworks proposing that expectations may influence symptom perception, while experiencing physical symptoms reinforces expectations over time.^15^.

Several mechanisms may potentially underlie these associations. Expectations are shaped by information and social modeling, both of which are highly salient in early pregnancy due to exposure to social networks and social media.^46,47^ Particularly in early pregnancy, when many women initially conceal their pregnancy in light of the high risk of miscarriage and only make limited use of their social resources,^1^ social media can become the primary information source.^48^ Women who selectively seek particularly negative information about pregnancy and childbirth might form more negative expectations and have higher prenatal worry levels.^49^ Likewise, observing symptom experiences from other pregnant women in their social network could impact their own symptom perception. Although we did not explicitly assess social media use and social observation in our study, robust evidence from the expectation literature^47^ supports the assumption that these factors likely shape expectations of what to expect in pregnancy.

In addition, learned or conditioned placebo and nocebo responses shape symptom perception through modulating physiological, immune, and neuroendocrine responses.^24^ In pregnancy, symptoms could be reinforced through learned associations of symptom experiences with situational cues such as tastes, odours, or places, for example repeatedly getting nauseous on the train. This is comparable to anticipated nausea and vomiting in chemotherapy.^21^ While this conclusion seems valid based on parallel evidence, experimental confirmation of these mechanisms in pregnancy is still pending.

Further, the bidirectional association of expectations and symptoms over time shown in our cross-lagged panel model may be consistent with a reinforcing cycle in which expectations are linked to symptom perception, while experiencing symptoms may in turn reinforce expectations.^50^.

Expectations may influence symptom appraisal, attentional processes and affect, thereby influencing symptom burden in pregnancy. Notably, women attempting to conceive perceive pregnancy-related symptoms earlier, sometimes even if they are not pregnant.^51^ Heightened attention to bodily signals might be driving this process.^41^ This is in accordance with the predictive processing framework that understands symptoms as the result of a complex interference process that may be influenced more by implicit expectations, prior experience, and affective states than by actual somatosensory input.^52^ This implies that expectations, negative affective states, and dysfunctional attention processes might influence the perception of pregnancy-related symptoms in interaction with hormone levels and other biological factors.^16,53^ It can be assumed that expectations focus the attention, so that pregnancy symptoms are perceived earlier. However, sensitivity and attention to interoceptive signals, assessed with the ISAQ,^41^ did not predict pregnancy-related symptom burden in our study, although it was associated with baseline symptom-related expectations. As the ISAQ captures interoceptive processes more broadly, it may lack specificity for pregnancy-related attentional mechanisms.

Pregnancy-related mindsets were meaningfully associated with symptom outcomes. Viewing the body as capable of coping with pregnancy was associated with lower symptom burden and disability, consistent with evidence from other medical contexts showing that adaptive body mindsets are linked to better well-being and are modifiable through targeted interventions.^31,54^ In contrast, perceiving pregnancy symptoms as reassuring signs was associated with higher symptom burden. Interpreting symptoms as indicators of a healthy pregnancy may increase attentional focus on bodily sensations, thereby amplifying symptom perception.^41^ Possibly, aggregating symptoms into a single evaluative mindset may obscure important differences between symptom types, as some symptoms (e.g., nausea) may carry positive connotations, whereas others (e.g., pain) may not. Future qualitative research is needed to better characterize pregnancy-specific symptom and body mindsets and to clarify how women interpret and attend to different symptoms during early pregnancy.

Strengths of this study include the prospective design, early assessment in the first trimester, preregistration, and converging analytic approaches. However, several limitations must be acknowledged. First, hCG levels could not be measured in our online design. Future studies should assess hCG levels to determine their impact and interaction with psychological factors.

Second, pregnancy after fertility treatment was overrepresented with 20.3%, as compared to around 2% of pregnancies after IVF in the population, leading to a potential selection bias. In addition, the sample was predominantly German, highly educated, and mostly living in relationships. Participants were partly recruited via social media and online pregnancy forums. Together with the overrepresentation of pregnancies after fertility treatment, this may limit the generalizability of the findings to broader and more socioeconomically or culturally diverse populations.

Third, several measurement issues need to be acknowledged. Due to a technical problem in the online survey, experience with previous pregnancies was partly invalid and could not be included in the prediction analyses. This represents an important limitation, as previous pregnancy and birth experiences may influence symptom expectations, symptom appraisal, and pregnancy-related distress.^50^ They should therefore be examined in future longitudinal studies. In the absence of a validated German version, the PSI was translated for the present study, and the PSI-expect measure was adapted from the PSI to assess expected symptom burden. Neither measure had been formally validated in German pregnant samples prior to this study. However, the PSI-expect closely resembles existing measures assessing expected symptoms and may therefore be considered to have preliminary validity.^55^ Last, the exploratory pregnancy-related mindset items were newly developed and were not based on a formal item development or validation process. Although most measures showed acceptable to good internal consistencies in the present sample, the two ISAQ subscales showed comparatively lower reliability. Findings involving these measures should therefore be interpreted cautiously and require further validation.

In addition, the follow-up assessment window in the second trimester was relatively broad, ranging from gestational weeks 13 to 20. Although most participants (96%) completed the assessment between weeks 13 and 16, and gestational week was not significantly associated with second-trimester symptom burden or disability, variation in gestational timing may nevertheless have influenced symptom burden levels.

Clinically, our findings suggest that symptom expectations and mindsets are important correlates of symptom burden and disability in early pregnancy. Importantly, expectations and mindsets are modifiable, and effective strategies for targeting them have been established in other medical contexts.^56,57^ Expectation-focused interventions have demonstrated benefits for improving both expectations and clinical outcomes in pain treatment,^58^ cardiac surgery,^59^ the management of side effects of breast cancer treatment,^55^ among others.^57^ Similarly, mindset interventions have successfully targeted symptom mindsets about side effects of antirheumatic treatment^60^ and oral immunotherapy,^61^ as well as body mindsets in cancer.^54^.

On this basis, interventions aimed at modifying negative pregnancy-related symptom expectations and mindsets could be developed for women who hold particularly maladaptive beliefs. Promising approaches include clear and empathetic communication strategies that normalize common pregnancy symptoms, manages attention to bodily sensations, offers positive framing of symptoms, and fosters adaptive mindsets.^29,56,60^ Body mindsets, such as perceiving the body as capable of carrying the pregnancy, may represent a particularly promising target, given their association with symptom burden and disability. Information about common pregnancy-related symptoms should be balanced with practical symptom coping strategies to enhance perceived control and self-efficacy. Importantly, focusing on changeable expectations should not imply shifting responsibility onto pregnant women themselves, rather, such approaches may offer an additional pathway to a sense of agency and control during pregnancy.^29^.

While sound evidence shows that the mental and physical health of mothers and offspring can be improved through psychological interventions in the second half of the pregnancy and peripartum,^62^ there is a lack of clear evidence regarding effective symptom management approaches in early pregnancy.^63^ While a few studies have shown benefits of psychological therapy,^64,65^ the potential of expectation management strategies has not been explored in pregnancy. Intervening early in pregnancy may help interrupt potentially reinforcing associations between expectations and symptom burden, which might have a favourable impact on pregnancy outcomes.^34^.

In terms of research implications, both qualitative and quantitative evidence is needed to identify relevant adaptive and maladaptive expectations and mindsets in early pregnancy, and explore women’s actual needs for interventions. Furthermore, the long-term impact of early pregnancy-related expectations on further pregnancy outcomes needs to be explored. Online interventions could be a promising approach given that women often have not disclosed their pregnancy or attended a gynaecological appointment at the time negative expectations might already come into play.

In conclusion, women’s expectations may represent an important psychological correlate of early pregnancy-related symptom burden and well-being. Addressing expectations and mindsets may represent a promising avenue for improving interdisciplinary care in early pregnancy.

## Supplementary Information

Below is the link to the electronic supplementary material.

Supplementary Material 1

## Data Availability

The research data used and analysed in this study will be shared by the corresponding author upon reasonable request.

## Declarations

**of generative AI and AI-assisted technologies in the writing process**.

During the preparation of this manuscript, the authors used DeepL and ChatGPT to support language refinement and improve readability. All content was subsequently critically reviewed and edited by the authors, who take full responsibility for the integrity and accuracy of the published work.
