# Supplementary material for: Expectations predict early pregnancy-related symptom burden
Source: Sci Rep. 2026 Jun 16;16:18705. doi: 10.1038/s41598-026-57961-w (PMC13272805; doi:10.1038/s41598-026-57961-w)
Supplement: Supplementary file 1 — Supplementary Material 1 [file 41598_2026_57961_MOESM1_ESM.docx]

# Supplement

### Table S1. Descriptive characteristics and internal consistencies of outcome and predictor measures

| Scale | Mean (SD), range | Internal consistency (Cronbach’s α) |
| --- | --- | --- |
| **Baseline first trimester** |  |  |
| Pregnancy-related symptoms (PSI) | 11.59 (4.55), 0–23 | .75 |
| Symptom expectations (PSI-expect) | 16.36 (8.61), 1–46 | .88 |
| Depressive symptoms (PHQ-9) | 8.69 (4.28), 0–23 | .77 |
| Pregnancy-related anxiety (PRAQ) | 6.16 (2.38), 2–10 | .83 |
| Self-efficacy (SWOP-K9) | 2.86 (0.49), 1.60–4.00 | .71 |
| Optimism (SWOP-K9) | 2.94 (0.67), 1.00–4.00 | .75 |
| Pessimism (SWOP-K9) | 1.96 (0.64), 1.00–3.50 | .65 |
| Interoceptive attention (ISAQ) | 3.12 (0.70), 1.25–4.75 | .60 |
| Difficulty disengaging from symptoms (ISAQ) | 2.70 (0.69), 1.25–4.50 | .49 |
| **Second trimester** |  |  |
| Pregnancy-related symptom burden (PSI) | 18.01 (7.71), 2–40 | .82 |
| Symptom-related disability (PDI) | 25.87 (13.49), 0–59 | .86 |

*Note*. n = 227.

### Table S2. Bivariate correlations between predictors in the first trimester and outcomes at the beginning of the second trimester

| Predictors | Pregnancy-related symptom burden (PSI) | Symptom-related disability (PDI) |
| --- | --- | --- |
| **Sociodemographic variables** |  |  |
| Age | -.10 (p = .133) | -.12 (p = .084) |
| In a relationship | .01 (p = .923) | .04 (p = .577) |
| Education ≥ 12 years | -.07 (p = .265) | -.04 (p = .548) |
| Professional status employed | -.07 (p = .324) | -.01 (p = .851) |
| Current sick leave | .13 (p = .844) | .07 (p = .292) |
| **Pregnancy-related factors** |  |  |
| Pregnancy week | .08 (p = .243) | .02 (p = .723) |
| Planned pregnancy | -.12 (p = .067) | -.07 (p = .306) |
| Fertility treatment | .04 (p = .585) | .07 (p = .289) |
| History of miscarriage(s) | .03 (p = .633) | .04 (p = .564) |
| Positive attitudes towards pregnancy | **-.18 (p = .005)** | **-.21 (p = .002)** |
| Feeling well informed about pregnancy | **-.26 (p < .001)** | **-.16 (p = .017)** |
| Medication intake | -.01 (p = .850) | .10 (p = .124) |
| Medication intake that can affect pregnancy | -.02 (p = .718) | .08 (p = .223) |
| **Psychological factors** |  |  |
| Depressive symptoms (PHQ-9) | **.49 (p < .001)** | **.43 (p < .001)** |
| Pregnancy-related anxiety (PRAQ) | **.28 (p < .001)** | **.16 (p = .016)** |
| Self-efficacy (SWOP-K9) | **-.25 (p < .001)** | **-.14 (p = .033)** |
| Optimism (SWOP-K9) | -.09 (p = .179) | **-.17 (p = .012)** |
| Pessimism (SWOP-K9) | **.21 (p = .001)** | **.14 (p = .034)** |
| Interoceptive attention (ISAQ) | .11 (p = .094) | .12 (p = .082) |
| Difficulty disengaging from symptoms (ISAQ) | -.09 (p = .196) | -.08 (p = .242) |
| **Symptom-related factors** |  |  |
| Pregnancy-related symptoms (PSI) | **.60 (p < .001)** | **.36 (p < .001)** |
| Proneness to symptoms | **.16 (p = .019)** | .06 (p = .383) |
| **Symptom expectations** |  |  |
| Symptom expectations (PSI-expect) | **.67 (p < .001)** | **.45 (p < .001)** |

*Note*. n = 227. Bivariate correlations (Pearson) for continuous data and point–biserial correlations for dichotomous data were calculated.

### Table S3: Bivariate correlations of pregnancy-related mindsets and expectations with pregnancy-related symptom burden and disability

|  | T1 Mindset: pregnancy symptoms as reassuring signs | T1 Mindset: body as capable | T1 Pregnancy-related symptoms (PSI) | T1 Symptom expectations (PSI-expect) | T2 Pregnancy-related symptom burden (PSI) | T2 Symptom-related disability (PDI) |
| --- | --- | --- | --- | --- | --- | --- |
| **T1 Mindset: pregnancy symptoms as reassuring signs** | **-** | -0.10  (p = 0.134) | **0.21  (p = 0.001)** | **0.18  (p = 0.006)** | **0.20 (p = 0.002)** | 0.11 (p = 0.092) |
| **T1 Mindset: body as capable** | -0.10  (p = 0.134) | **-** | **-0.17  (p = 0.009)** | **-0.26  (p = < .001)** | **-0.21  (p = 0.001)** | **-0.20 (p = 0.003)** |
| **T1 Pregnancy-related symptoms (PSI)** | **0.21  (p = 0.001)** | **-0.17  (p = 0.009)** | **-** | **0.51  (p = < .001)** | **0.60  (p = < .001)** | **0.36  (p = < .001)** |
| **T1 Symptom expectations (PSI-expect)** | **0.18  (p = 0.006)** | **-0.26  (p = < .001)** | **0.51  (p = < .001)** | **-** | **0.67  (p = < .001)** | **0.45  (p = < .001)** |
| **T2 Pregnancy-related symptom burden (PSI)** | **0.20  (p = 0.002)** | **-0.21  (p = 0.001)** | **0.60  (p = < .001)** | **0.67  (p = < .001)** | **-** | **0.52  (p = < .001)** |
| **T2 Symptom-related disability (PDI)** | 0.11  (p = 0.092) | **-0.20  (p = 0.003)** | **0.36  (p = < .001)** | **0.45  (p = < .001)** | **0.52  (p = < .001)** | - |

*Note*. n = 227. T1 refers to the first-trimester assessment; T2 refers to the assessment at the beginning of the second trimester. Bivariate correlations (Pearson) were calculated.
